# Healthcare leadership effectiveness among managers in Public Health institutions of Addis Ababa, Central Ethiopia: a mixed methods study

**DOI:** 10.1186/s12913-022-07879-6

**Published:** 2022-04-22

**Authors:** Kiros Teame, Ayal Debie, Mikiyas Tullu

**Affiliations:** 1 Sub-City Health Office, Addis Ababa City Administration, Addis Ababa, Ethiopia; 2 Department of Health Systems and Policy, Institute of Public Health, College of Medicine and Health Sciences, University of Gondar, Gondar, Ethiopia; 3 School of Public Health, College of Health Sciences, Kotebe Metropolitan University, Addis Ababa, Ethiopia

**Keywords:** Healthcare, Leadership, Effectiveness, Managers, Addis Ababa, Ethiopia

## Abstract

**Background:**

Leadership is the ability to influence the attitudes, beliefs, and abilities of employees to achieve organisational goals. It is crucial for the successes or failures of organisational performance. Healthcare organizations need effective leadership to manage the health service delivery reforms efficiently and effectively. However, there was no adequate evidence on the current status of the healthcare leaders to make evidence-based decisions. Therefore, this study aims to assess the effectiveness of healthcare leadership and associated factors among managers working at public health institutions in Addis Ababa, Ethiopia.

**Methods:**

Institution-based cross-sectional study triangulated with the qualitative study was employed from 01 April to 01 June 2021. A total sample of 844 healthcare managers were used to assess their leadership effectiveness. Multi-stage sampling followed by a simple random sampling technique was used to select the participants. Binary logistic regression model was fitted to identify the factors associated with healthcare leadership effectiveness. Adjusted odds ratio (AOR) with 95% confidence interval (CI) and p-value less than 0.05 during multivariable logistic regression were used to declare the factors associated with the outcome variable. We conducted key informant interviews (KIIs) to explore the views of healthcare managers on their leadership practices, mainly on vision creation, developing followership and implementing vision. We also tape-recorded the KIIs and then transcribed word by word and finally translated it into English. We conducted a thematic analysis to supplement the quantitative findings.

**Results:**

In this study, 46.8% (95% CI: 43.4 -50.2) of the participants had effective healthcare leadership practices. Emotional intelligence (AOR = 7.86; 95% CI; 4.56, 13.56), democratic managers (AOR = 4.01, 95% CI; 1.98, 8.14), master or above education (AOR = 5.1; 95% CI; 2.07, 12.61) and work experience (AOR = 3.44, 95% CI; 1.24, 9.55) were positively associated with healthcare effective leadership. The challenges in healthcare leadership were mainly associated with lack of leadership knowledge and skills. In addition, autocratic leaders negatively influenced managers ability to work closely with the staffs and affected employee’s motivation. On the contrary, emotionally intelligent managers were effective on employee handling, providing chance to talk, understanding their feelings and needs.

**Conclusion:**

Healthcare managers had low capacity on vision creation, implementation and developing followership, particularly the ability of vision creation was very low. Lack of leadership knowledge and skills and frequent use of autocratic leadership were the challenges for healthcare leadership effectiveness. This could also negatively influence organisational performances, managers’ ability to work closely with the staffs and reduced employee’s motivation. Therefore, strengthening emotional intelligence and empowering managers will be very helpful to improve leading health cares.

**Supplementary Information:**

The online version contains supplementary material available at 10.1186/s12913-022-07879-6.

## Background

Health care is a complex industry and needs effective leadership to provide successful healthcare services [1, 2]. Healthcare leadership is the cross-cutting components of the health system building blocks, which helps to strengthen the other five building blocks [3]. Health system needs effective leadership to mobilise resources to maximise favorable health impacts [4, 5]. Leadership is the ability to influence the attitudes, beliefs, and abilities of employees to achieve their organisational goals [6]. Leadership is also vital and an influential factor to deliver high-quality, compassionate and patient-centered care through improving organisational performance to solve challenges of good governance [7, 8]. Effective leadership is critical for healthcare organisations to illustrate continuous reforms for efficient and effective service delivery at regional and global levels [9, 10]. Healthcare managers’ leadership capacity determined the health system’s ability to deliver quality healthcare services [11]. Effective leadership has also a paramount importance to manage new change initiatives and implement the national reforms within the health sectors [12, 13].

The African health system is characterised as fragile and weak associated with its poor leadership despite leadership plays a pivotal role for better performance of managers at Primary Health Care Units (PHCUs) [14, 15]. In Ethiopia, lack of leadership commitment is the main drawback to perform health reforms [12]. Unavailability of health workers associated with poor leadership can be the causes for loss of lives [16]. Accordingly, healthcare leadership assessments at five public hospitals of Addis Ababa only scored 61% [17]. Leadership effectiveness can be influenced by various organisational and personal factors, such as variations in gender, job commitments, work experiences, organisational cultures, leadership styles, and emotional intelligences (EI)[18–20]. An ineffective leadership on the contrary leads to low motivation, poor collaboration, mistrust, low self-confidence, and insecurities of workers [21].

Effective leadership has a significant positive impacts on overall organisational performance and leader's emotional regulation [22, 23]. Leadership capacity-building interventions among managers brought differences in healthcare performance [24]. Ethiopia has planned a health sector transformation plan (HSTP), which requires effective healthcare leadership to achieve its comprehensive vision and become a middle-income country (MICs) in the year 2025 [25]. Despite healthcare leadership was one of the pillars of excellence, lack of strong leadership in the health sector was the main challenges to realise HSTP in Ethiopia [26]. Moreover, there was no adequate evidences on leadership practices among healthcare managers in Ethiopia. Therefore, this study aims to assess healthcare leadership effectiveness and associated factors among managers working at public health institutions in Addis Ababa, Ethiopia.

## Methods and materials

### Study design and settings

An institution-based cross-sectional study triangulated with the qualitative study was conducted from 01 April to 01 June 2021. This study was conducted at public health institutions of Addis Ababa city administration. Addis Ababa is the capital city of Ethiopia and the diplomatic center of Africa. It has a total population of 5,005,524 in the 2021 world population review report [27]. Addis Ababa is located at the heart of Ethiopia at 9^o^ 2' N latitude and 38^o^ 45' E longitude. The average altitude of Addis Ababa is 2,400 m above sea level. The area of Addis Ababa also occupies a total of 540 km^2^ [28]. Addis Ababa city administration has 10 sub-cities, 117 woredas (districts), 101 public health centers, and 12 public hospitals.

### Population

All healthcare managers working at public health institutions of Addis Ababa city administration were the source population while those healthcare managers at selected public health institutions were the study population. All healthcare managers who were working for more than six months at public health institutions of Addis Ababa city administration but those managers on annual leave and absent at workplace during data collection period were excluded. Moreover, healthcare managers working at public hospitals, health centers and health offices were the study population for the qualitative study.

### Sample size and sampling procedure

Single population proportion formula was used to calculate the sample size. In addition, we considered 50% proportion, 5% margin of error, 10% non-response rate, 2 design effect and 10% non-response rate to determine the sample size. Thus, the final sample size was 844. Public health institutions in Addis Ababa City Administration were stratified as hospitals, health centers and health offices. The health offices include managers/ directors of sub-city health offices and Regional Health Bureau. We employed multi-stage stratified sampling technique to select study participants. Then, we used simple random lottery method to select 6 hospitals, 44 health centers and 6 health offices. Finally, the total sample size was proportionally allocated to each selected public health institutions based on their number of managers. Simple random sampling technique was used to select the participants.

We conducted KIIs to explore the views of healthcare managers on their leadership capability, mainly on their ability on vision creation, implementation of their vision and developing followership. Purposive sampling technique was used to select key informants (KIs) at public hospitals, health centers and health offices. We considered their position and managerial experience to select the KIs. We included 11 KIs to explore the barriers and facilitators of healthcare managers for leadership effectiveness.

### Data collection tools and procedures

Structured self-administered questionnaire and semi-structured interview guide were prepared through reviewing of different literatures [29–33]. Firstly, we collected the quantitative data using a self-administered questionnaire from healthcare managers working at public hospitals, health centers and health offices. The questionnaire includes socio-demographic characteristics, positions of managers, leadership effectiveness, substance use characteristics, and emotional intelligence. The healthcare leadership effectiveness of managers was measured using a questionnaire adapted from Management Research Group (MRG) [29, 30]. We invited experts to review each measuring item of effective leadership to check and approved the content validity of the tool. We also calculated Cronbach alpha (α) to check reliability of effective leadership measuring tool and we got Cronbach alpha (α) value of 0.78. Quick Emotional Intelligence Self-Assessment (QEISA) tool adapted from 2015 Paul Mohapel model was used to assess emotional intelligence of healthcare managers [31]. We also checked the reliability of emotional intelligence assessment tool through calculating Cronbach alpha (α) and we found the Cronbach alpha (α) value of0.81. The content validity was also checked and approved by leadership experts after reviewing each item of the assessment tool to measure emotional intelligence of managers.

Semi-structured interview guide was also prepared to collect the qualitative data. The interview guide, includes the description of leadership effectiveness, facilitators and obstacles/ barriers for leading health cares. We also probed the KIs responses to elaborate their perception on effective leadership, particularly with respect to vision creation, implementation of vision and developing followership perspectives. We first prepared the questionnaire and interview guide in English and translated to Amharic and back to English to ensure its consistency. One day training on the basic techniques of data collection was given to data collectors and supervisors before the actual data collection period. Seven Bachelor of Science (BSc) clinical nurses and three BSc in Public Health who had prior data collection experiences were recruited for data collectors and supervisors, respectively. Data collectors were also briefed on each item included in the data collection tool. We pre-tested the questionnaire on 42 healthcare managers (5% of the sample size) at Tirunesh Beijing hospital, Arada sub-city health office, and woreda 17 health center before the actual data collection period. We also pre-tested the interview guide among three managers working at these selected health facilities. Necessary modification was also done for both the quantitative questionnaire and qualitative interview guide based on the pre-test findings. Initially, we informed the KI participants about the aim, risks and benefits of the study and we received their permission. The principal investigator collected using a semi-structured interview guide. The interview process was taken 15–20 min and conducted through maintaining the KIs privacy. The whole discussions of the KII process were tape-recorded and notes were also taken. The interviewer probed the interviewees following their response to explore more information. Supervisors and principal investigator checked the completeness and consistency of data on a daily basis.

### Variables and measurements

Leadership effectiveness was the outcome variable while socio-demographic characteristics (age, gender, salary, family size, work experience, education, type institutions, emotional intelligence, position of managers, leadership style and substance use characteristics of managers were the independent variables. We used three dimensions, namely vision creation, implementation of vision, and developing followership; and we had a total of 15 items to measure healthcare leadership effectiveness. Each item was measured using a five-point Likert scale (1 = not at all, 2 = once in a while, 3 = sometimes, 4 = fairly often and 5 = always). As a result, the total score could be ranged 15–75, and then we used demarcation threshold formula: ((total highest score—total lowest score) / 2 + total lowest score)/ total highest score * 100% [34] to categorise the level of leadership effectiveness as “high” and “low”. As a result, we categorised healthcare managers who scored over 60% and ≤ 60% as having high and low effective leadership practices, respectively. Emotional intelligence is a capacity to understand the emotions of one-self and/ or others to distinguish between them and exercise in guiding one's thoughts and practices [35]. We had four dimensions to assess emotional intelligence, namely self-awareness, emotional management, social awareness, and relationship management; and we had also a total of 40 self–reporting items to measure EI [36]. Each item also contained a 5-point Likert scale (4 always to 0 never). We categorised managers’ emotional intelligence as low, moderate, and high if managers scored < 53, 53–106 and > 106, respectively [37]. The unit of measurement for the salary of healthcare managers was Ethiopian Birr (ETB), but we changed it into United States Dollar (US$). We used the exchange rate of ETB to US$ during in April 2021 (1US$ = 41.47 ETB).

### Data management and analysis

Data were entered into Epi data version 4.4.1 and exported to SPSS version 20 for analysis. Descriptive statistics were reported using texts, graphs and tables. Binary logistic regression model was fitted to identify factors associated with effective healthcare leadership. Variables with *p*-value < 0.2 during the bivariable analysis were entered into multivariable logistic regression. Adjusted odds ratio (AOR) with 95% confidence interval (CI) and p-value less than 0.05 were used to declare the factors associated with the outcome variable and the strength of association. For the qualitative part, the raw data were collected in the form of field notes and tape recording. The tape-recorded was transcribed and translated into English language for analysis. We coded the translated texts using manual text markers to generate themes based on the ideas of the key informants. Thematic analysis and narrative synthesis were done to synthesise the qualitative findings.

## Results

### Socio-demographic characteristics

A total of 818 managers participated in the study with a response rate of 96.9%. More than half (52.3%) of the participants were males and over forty percent (45.4%) of managers also aged between 25—30 years. Majority (62.5%) of the healthcare managers had Bachelor of Science (BSc) or Bachelor of Art (BA) Degree. Accordingly, 54% of managers were case team coordinators and had a monthly salary of US$ 188.11–264.84. Nearly one-tenth of the managers had 15 or more years work experience and two-thirds of the participants were working at health centers. About eight percent (7.5%) of the participants had seven or more household members (Table 1). On top of that, we included a total of 11 KIs (two from health offices, six from health centers and three from hospitals) to explore the views of healthcare managers on effective leadership. Three of our KIs were females. The professional characteristics of our KIs were also medical doctor (1), pharmacist (1), biomedical science (1), and the rests were nurses and public health officers.

**Table 1:** Socio-demographic characteristics of managers working in public health institutions of Addis Ababa, 2021 (*n* = 818)

Variables	Category	Ferqency	Percent (%)
Gender	Male	428	52.3
	Female	390	47.7
Age in years	25–30	371	45.4
	31–35	245	30.0
	36–40	116	14.2
	≥ 41	86	10.5
Salary (US$)	≤ 126.60	45	5.5
	126.62–188.09	247	30.2
	188.11–262.84	440	53.8
	≥ 262.86	86	10.5
Educational status	Diploma	81	9.9
	First Degree	511	62.5
	Masters or above	226	27.6
Work experience in years	1–5	225	27.5
	6–10	431	52.7
	11–15	82	10.0
	≥ 16	80	9.8
Family size	≤ 3	458	56.0
	4–6	299	36.6
	≥ 7	61	7.5
Position	Case-team coordinator	444	54.3
	Sub-core process coordinator	97	11.9
	Core process coordinator	210	25.7
	Others	67	8.2
Institution type	Health center	571	69.8
	Hospital	190	23.2
	Health offices	57	7.0

*1US$* = *41.47 ETB*

### Emotional intelligence, leadership style, substance use, and level of managers

In this study, nearly half (47.1%) of healthcare managers in the study area had moderate level of emotional intelligence. More than fifty-five percent (57.2%) of the healthcare managers were low-level healthcare managers. More than two-thirds (64.7%) of healthcare managers exercised democratic leadership styles. Just two-thirds (67.4%) of healthcare managers did not use any substances (Table 2).

**Table 2:** Emotional intelligence, managerial level and substance use among health institutions managers in Addis Ababa, 2021 (*n* = 818)

Variables	Category	Frequency	Percent (%)
Leadership style	Democratic	531	64.9
	Autocratic	221	27.0
	Laissez-faire	66	8.1
Emotional intelligence	Low	244	29.8
	Moderate	385	47.1
	High	189	23.1
Substance use	Yes	267	32.6
	No	551	67.4
Drink alcohol (*n* = 267)	Yes	263	98.5
	No	4	1.5
Chew Khat (*n* = 267)	Yes	12	4.5
	No	255	95.5
Smoke cigarettes (*n* = 267)	Yes	20	7.5
	No	247	92.5

### Leadership effectiveness

In this study, 46.8% (95% CI: 43.4–50.2) of healthcare managers had exercised effective healthcare leadership, particularly with respect to creation of vision, implementation of their vision and developing of followership. Accordingly, 43.5, 53.4 and 53.1% of managers had better leadership practices on creation of vision, implementation of their vision, and development of followership, respectively. Over seventy percent (72.2%) of participants had good ethical values and act consistently. Two-thirds (67.7%) of managers were working on a daily basis to achieve their organisational vision. Over half (53%) of the mangers had also poor experiences on preparing future leaders and 42.8% of managers had poorly communicating their mission and vision (Table 3).

**Table 3:** Proportion of healthcare leadership effectiveness among managers at public health institutions Addis Ababa, 2021 (*n* = 818)

Items	Not at all (1)	Once in a while (2)	Sometimes (3)	Fairley often (4)	Always (5)	Proportion of managers (%)	
						Low effectiveness (scored < 3)	High effectiveness (scored ≥ 3)
**Create a vision**							
I study problems in light of past practices to make sure predictability, trengthen the status quo and minimize risk	14.8	24.6	37.5	12.1	11.0	39.4	60.6
I feel comfortable in fast-changing environments; being willing to take risks and to consider new and untested approaches	15.6	26.7	36.4	12.7	8.6	42.3	57.7
I set a clear vision for my sector	14.4	25.4	35.7	14.3	10.1	39.9	60.1
I communicate the mission and vision of the sector with people around me	14.4	28.4	27.5	16.6	13.1	42.8	57.2
Taking a long-range, broad approach to problem-solving and decision making through objective analysis, thinking ahead, and planning	13.3	18.8	37.4	21.4	9.0	32.2	67.8
**Develop followership**							
I am strongly persuasive and assertive stance to convince my followers	16.1	20.9	35.1	18.1	9.8	37.0	63.0
I am emotionally expressive and reactive	16.4	17.4	40.5	21.0	4.8	33.7	66.3
I am ready to develop tomorrow’s leaders	19.3	33.7	25.2	16.5	5.3	53.0	47.0
I act in an extroverted, friendly, and informal manner to show a capacity to quickly establish free and easy interpersonal relationships	15.8	28.1	24.3	20.9	10.9	43.9	56.1
I have ethical values & act consistently	11.1	16.6	22.7	32.3	17.2	27.8	72.2
**Implement vision**							
I adopt a systematic and organized approach; preferring to work in a precise, methodical manner; developing and utilizing guidelines and procedure	13.8	22.4	32.8	22.9	8.2	36.1	63.9
I work on a day-to-day basis for achieving the sector vision	10.5	21.8	29.6	27.6	10.5	32.3	67.7
I emphasize the production of immediate results by focusing on short-range and practical strategies	15.5	18.9	32.0	25.7	7.8	34.5	65.5
I state clearly what I want and expect from others; clearly, express my thoughts and ideas; maintaining a precise and constant flow of information	11.9	18.6	34.6	26.0	8.9	30.5	69.5
I capacitate others by giving them important activities and sufficient autonomy to exercise their own judgment	15.9	19.8	32.3	25.8	6.2	35.7	64.3

### Factors associated with effective healthcare leadership

We fitted binary logistic regression model to identify the factors associated healthcare managers leadership effectiveness. Those independent variables with p-value of less than 0.2 during bivariable analysis were entered into multivariable analysis. As a result, we included age, education, experiences, salary, family size, institution, level of managers, leadership style, and emotional intelligence of healthcare managers during multivariable analysis.

The odds of effective leadership among healthcare managers attended master or above education were 5.1 times (AOR = 5.1; 95% CI; 2.07, 12.61) higher compared with diploma holders. This finding was supported by the qualitative study in which a 58 years old nurse working at the hospital reported that educational status of healthcare managers has an impact on leadership effectiveness to realise the goal of the health sectors. “I think the level of education of healthcare managers may affect their ability to lead the health sectors. Since more educated healthcare managers might have higher knowledge of leadership. This can help managers to do better job by turning their knowledge into action” (a 58 years old nurse at the hospital).

A 44 years old public health officer working at the health center on the other hand replied that poor knowledge and skills of managers has an impact on leadership effectiveness. “The challenges in healthcare leadership effectiveness are mainly associated with managers’ lack of knowledge and skills on leading healthcare. This might be associated with poor supportive supervision, weak monitoring and evaluation of their performance due to lack of confidence, which in turn reduce to achieve the health sector goals” (a 44 years old public health officer at the health center).

The odds of effective leadership among healthcare managers who had work experience more than 15 years were 3.4 times (AOR = 3.44, 95% CI; 1.24, 9.55) higher than their counterparts. This finding was also inline with our KIIs findings, which indicated more experienced managers were effective in leading health cares. “Experiences of healthcare managers are critical for healthcare leadership effectiveness because managers can provide better leadership through identifying their strengths and weaknesses from their previous exposures and performances. This can help managers to take early corrective measures which enables them to lead effectively” (a 30 years old public health officer at the health center). “Work experiences of healthcare managers influence leadership effectiveness since someone works on the same position for certain years, the level of their concern on what to do and how to do may not their issues. As a result, this might help managers to do things easily in an effective way” (a 40 years old psychiatric nurse in the hospital).

On the other hand, a 30 years old public health officer working at the health center reported that managers who had low work experience might not be effective on leading health cares. “Managers who had a lesser work experience might bother about how to lead effectively, which can influence their leadership effectiveness” (a 30 years old public health officer at the health center).

A 37 years old public health officer working at the hospital also stated that age of healthcare managers might also affect the healthcare leadership effectiveness. “I think that age of healthcare managers has an impact on health sector performance because they would have better understood how to work effectively. This can be associated with older healthcare managers had got better experience on how to influence and direct staff members towards organisational goals” (a 37 years old public health officer working at the hospital).

The odds of effective healthcare leadership among democratic healthcare managers were 4 times (AOR = 4.01,95% CI;1.98, 8.14) higher compared with laissez-faire managers. This was also supported by a medical doctor at the hospital indicated that democratic leaders are more preferable for leadership effectiveness. “Democratic leaders are effective on organisational performance since they tried to promote and initiate employees to search problem-solving strategies/ ideas and reduce institutional disputes which helps them to reach at an advanced output” (a 54 years old medical doctor working at the hospital).

A 28 years old pharmacist working at the health center also reported that healthcare managers leadership can negatively affect leadership effectiveness and organisational performance. “Leadership style has an impact on the effectiveness of healthcare managers. I think it is important to use all three different leadership styles as necessary for the situation that fits for effectiveness; however, our manager has been exercised autocratic leadership style most dominantly, which may have negative impact on the performance of our organisation” (a 28 years old pharmacist in the health center).

“The leadership approach that we followed in our hospital is usually commanding. This may affect to work closely with the staffs and reduce healthcare performances since the employees are not motivated to do their job. This kind of leadership style may also suitable or appropriate for the team sprite of employees” (a 54 years old medical doctor in the hospital).

The odds of effective healthcare leadership among high emotional intelligent managers were 7.8 times (AOR = 7.86; 95% CI; 4.56,13.56) higher than compared with low emotional intelligent managers. A 29 years old nurse at the health center also stated that emotional intelligent managers are vital for proficient and evidence-based decision-making. “Emotional intelligent managers affect on leading health cares since a manager has better understood and handle the employees’ emotions that will help employees to perform with interests in an effective manner” (a 29 years old nurse at the health center). On the contrary, three KIs working at the health center and health offices reported that poor emotional intelligent managers can negatively affect their effectiveness in leading health cares. “Managers with low emotional intelligence can affect their effectiveness because those kinds of managers may decide without considering and understanding the situations of employees” (a 29 years old nurse at the health center). “Health office managers may not let you give a chance to talk and they do not want to understand the feelings and needs of their employees. They usually say that I know it and they do not want to listen anyone” (a 50 years old public health officer at health office). “Healthcare managers are not currently emotionally intelligent since they are politically motivated. I think that managers, particularly who are working at the healthcare sectors can not create and introduce new initiatives/ strategies to change. They do not analyse the feelings of their followers rather they mainly focused on to be loyal to their supervisors and political view” (a 24 years old Biomedical technician at the health office).

A 55 years old nurse working at the health center stated that substance use and personal interests of managers influenced their leadership effectiveness. “Substance use can affect managers’ leadership effectiveness because managers might not be focused on doing their work and this can prevent them to perform their duties and responsibilities” (a 55 years old nurse at the health center).

A 40 years old psychiatric nurse working at the hospital also stated that managers who are working for their personal gain might not be worried on changing their institution. “Healthcare managers are working for their personal gain might not be worried on changing their institution. As a result, they are not committed to do their tasks with full transparency and accountability, which may lead to poor healthcare performance associated with poor leadership” (a 40 years old psychiatric nurse at the hospital) (Table 4).

**Table 4:** Factors associated with healthcare leadership effectiveness among managers in public health institutions in Addis Ababa, Ethiopia, 2021 (*n* = 818)

Variables	Category	Leadership effectiveness		COR (95% CI)	AOR (95% CI)	*P*-value
		High	Low			
Age in years	25–30	134	237	1	1	
	31–35	118	127	1.64 (1.18,2.28)	1.10 (0.67,1.79)	0.712
	36–40	68	48	2.51 (1.64,3.84)	0.74 (0.37,1.50)	0.409
	≥ 41	63	23	4.85 (2.88,8.17)	0.64 (0.25,1.66)	0.361
Education	Diploma	13	68	1	1	
	First Degree	202	309	3.42 (1.84,6.35)	2.22 (1.02,4.85)	0.046
	Master’s and above	168	58	15.15 (7.80,29.44)	5.10 (2.07,12.61)	0.000
Experience in years	1–5	58	167	1	1	
	6–10	200	231	2.49 (1.75,3.55)	1.60 (1.03,2.49)	0.037
	11–15	60	22	7.85 (4.43,13.92)	3.96 (1.77,8.85)	0.001
	≥ 16	65	15	12.48 (6.61,23.57)	3.44 (1.24,9.55)	0.018
Salary (US$)	≤ 126.60	16	29	1	1	
	126.62–188.09	73	174	0.76 (0.39,1.48)	0.59 (0.24,1.41)	0.232
	188.11–262.84	223	217	1.86 (0.98,3.53)	0.63 (0.26,1.52)	0.301
	≥ 262.86	71	15	8.58 (3.76,19.60)	1.64 (0.50,5.36)	0.411
Family size	≤ 3	172	286	1	1	
	4–6	173	126	2.28 (1.70,3.07)	1.12 (0.72,1.77)	0.613
	≥ 7	38	23	2.75 (1.58,4.77)	0.73 (0.33,1.63)	0.439
Institution	Health Center	244	327	1	1	
	Hospital	94	96	1.31 (0.94,1.82)	0.68 (0.42,1.10)	0.113
	Health Office	45	12	5.03 (2.60,9.70)	1.28 (0.55,2.97)	0.572
Level of managers	Top	36	5	12.17 (4.69,31.58)	2.09 (0.64,6.80)	0.602
	Middle	173	136	2.15 (1.60,2.88)	1.19 (0.72,1.96)	0.255
	Lower	174	294	1	1	
Leadership Style	Democratic	335	196	4.23 (2.41,7.41)	4.01 (1.98,8.14)	0.000
	Autocratic	29	192	0.37 (0.19,0.72)	0.46 (0.21,0.99)	0.048
	laissez-faire	19	47	1	1	
Emotional intelligence	Lower	73	171	1	1	
	Moderate	165	220	1.76 (1.25,2.47)	2.07 (1.35,3.15)	0.001
	High	145	44	7.72 (4.99,11.92)	7.86 (4.56,13.56)	0.000

*COR Crud Odds Ratio, AOR Adjusted Odds Ratio, CI Confidence Interval, 1US$* = *41.47 ETB*

## Discussion

This study revealed that healthcare managers leadership effectiveness and associated factors among managers in public health institutions. Factors associated with effective leadership were educational status, managerial position, emotional intelligence, and leadership style. In this study, the application of healthcare managers’ leadership effectiveness, particularly managers ability of vision creation was low. We also found that the challenges in healthcare leadership are mainly associated with managers’ lack of leadership knowledge and skills. Autocratic leaders also negatively influence organisational performances and affect managers to work closely with the staffs and reduce employee’s motivation. Substance use may also affect managers’ work performances and prevents them from realising their duties and responsibilities. On the contrary, emotionally intelligent managers are effective on employee handing, providing chance to talk, understanding their feelings and needs.

In this study, 46.8% (95% CI: 43.4–50.2) of public healthcare managers had effective leadership. Accordingly, public healthcare managers had 43.5%, 53.4% and 53.1% better experiences on creation of vision, implementation of vision, and developing followership, respectively. The finding was higher than studies conducted in North Shoa zone (31.6%) and GIFT group companies (32%) [38] in Ethiopia. However, it is lower than a study conducted in Sri Lanka (74%) [39]. The possible justification for this discrepancy might be due to the differences in the study area. The current study was done at the capital city of Ethiopia. This may positively influence the healthcare leadership effectiveness associated with the availability of infrastructures/ other resources within the healthcare facilities. Sociodemographic characteristics of the healthcare managers, such as age, experience and educational level could be also other reasons for the discrepancy. This is because more experienced and educated healthcare managers might be concentrated at the capital city of the nations.

The odds of effective healthcare leadership among managers who attended master or above education were higher compared with managers who had diploma. This study finding was inline with studies conducted in Ethiopia, South Africa and India [40–42]. This might be associated with managers who attended higher education might have good capability of better leadership practices. This finding could also be supported by the theory of planned behavior, which attitude and behavioral changes could convert the attitude to effective task performance by controlling other bottle necks [43].

The odds of effective leadership among managers who had more experiences were higher compared with managers who had less experiences. This study finding was consistent with studies conducted in Ethiopia and Nigeria [44, 45]. This could be also supported by social learning theory, which self-efficacy of the managers might help them to improve their behavioral capability to effectively perform their job with more exposure (observation) of their working environment [46, 47]. The possible justification for this could be managers who had more experiences were effective in leadership because managers might have more exposure and skills to solve various problems on their job.

The odds of effective leadership among democratic managers were higher compared with managers who practiced laissez-faire leadership style. This study finding was supported by studies conducted in Malaysia, Iran and Nigeria [48–50]. This could also be supported by behavioral leadership theory, which depicts that managers who manifest democratic leadership behavior leads to increase organizational performance in relation with employees participation and motivation [51]. The possible justification could be due to managers who exercised democratic leadership could be responsive for participatory decision-making and responsible management.

The odds of effective leadership among high emotional intelligence managers were higher compared with managers with low emotional intelligence. This finding was inline with the studies conducted in Kaffa zone, Addis Ababa and Gaza strip in Palestine [29, 52, 53]. The finding was also consistent with the studies in Egypt, Australia, Pakistan and India [54–56]. This could be supported by the theory of performance, which elucidates managers who had self-awareness, self-management, social-awareness and relationship-management competencies of emotional intelligence make an organizational climate to be suitable for effective performance [57].

### Strengths and limitations

This study assessed healthcare managers’ performance/ application in terms of vision creation, developing of followership and implementation of vision. This study was also explored the views of managers using qualitative study. In addition, the finding will help to design intervention strategies from the barriers, scale up the facilitators, and implement different initiatives and/ or projects to capacity building and/or policy development in leading health cares. On the contrary, the study might prone to a response bias in relation with the managers’ self-assessment of their own effective leadership practices. This study, therefore, might not assess the perceptions of peers, subordinates, immediate supervisors and others on healthcare managers’ leadership practices. The study might not also show cause-and-effect relationship associated with its cross-sectional study design.

## Conclusion

Healthcare managers had low capacity on vision creation, implementation and developing followership, particularly the ability of vision creation was very low. Educational status, managerial position, emotional intelligence, and leadership style were the factors affecting healthcare leadership effectiveness. The challenges in healthcare leadership were mainly associated with managers’ lack of knowledge and skill on leadership. Autocratic leaders negatively influence organisational performances and managers ability to work closely with the staffs and reduce employee’s motivation. Emotionally intelligent managers are effective on employee handing, providing chance to talk, understanding their feelings and needs. Capacitating healthcare managers emotional intelligence shall improve their effectiveness in leading healthcare. More experienced and educated healthcare managers are also critical to enhance their leadership effectiveness. Further researches shall conduct on the subject including employees’ view on effective leadership. We will also recommend future researchers to assess the impact of substance use on leading health cares, particularly its effect on vision creation, implementation and developing followership.

## Supplementary Information

**Additional file1:** 

## Data Availability

Data will be available upon reasonable request from the corresponding author.

## Abbreviations

AARHB: Addis Ababa Regional Health Bureau
AOR: Adjusted Odds Ratio
BPR: Business Processing Reengineering
BSC: Balanced Score Card
CI: Confidence Interval
COR: Crude Odds Ratio
EI: Emotional Intelligence
HSTP: Health Sector Transformation Plan
LE: Leadership Effectiveness
MRG: Management Research Group
PHCU: Primary Health Care Units
SDGs: Sustainable Development Goals
UNRWA: United Nations Relief and Works Agency for Palestine Refugees
