# Supplementary material for: Healthcare leadership effectiveness among managers in Public Health institutions of Addis Ababa, Central Ethiopia: a mixed methods study
Source: BMC Health Serv Res. 2022 Apr 22;22:540. doi: 10.1186/s12913-022-07879-6 (PMC9034590; doi:10.1186/s12913-022-07879-6)
Supplement: Supplementary file 1 — Additional file1: [file 12913_2022_7879_MOESM1_ESM.docx]

Part I : Socio-demographic characteristics

| S.No. | Items | Categories | Skip | Code |
| --- | --- | --- | --- | --- |
| 101 | Sex | 1. Male 2. Female |  |  |
| 102 | Age in years | __________ |  |  |
| 103 | Level of Education | 1. Diploma 2. First degree 3. Master Degree 4. Medical Doctor (MD) 5. Philosophy of Degree (PhD) 6. Others (specify) ____________ |  |  |
| 104 | Work experience in years | _______ |  |  |
| 105 | Current work position | 1. Chief excutive officer 2. Medical director 3. Core process coordinator (team leader) 4. sub-core process coordinator (Sub-team leader) 5. Metron 6. Case-team coordinator 7. Sub-city health office head 8. Others (specify) ______________ |  |  |
| 106 | Type of current working institutions | 1. Hospital 2. Health Center 3. Sub-city health office (SCHO) 4. Addis Ababa Regional Health Bureau (AARHB) |  |  |
| 107 | Monthly salary in Ethiopian Birr (ETB) | ______________ |  |  |
| 108 | Family size in number | _____________ |  |  |

Part II: Position and characteristics of managers

| S. No. | Items | Coding categories | Skip to | Code |
| --- | --- | --- | --- | --- |
| 201. | In your organizational specific context, in which hierarchies (level) of managers are your position is categorized? | 1. Top 2. Middle 3. low |  |  |
| 202. | What type of leadership style did you exercise most dominantly? | 1. Democratic 2. Autocratic 3. Laissez faire |  |  |

Part III: Emotional intelligence assessment

| S.No | A. Self-Awareness | Never (0) | Rarely (1) | Some times (2) | Often (3) | Always (4) |
| --- | --- | --- | --- | --- | --- | --- |
| 301 | My feelings are clear to me at any given moment | 0 | 1 | 2 | 3 | 4 |
| 302 | Emotions play an important part in my life | 0 | 1 | 2 | 3 | 4 |
| 303 | My moods impact the people around me | 0 | 1 | 2 | 3 | 4 |
| 304 | I find it easy to put words to my feelings | 0 | 1 | 2 | 3 | 4 |
| 305 | My moods are easily affected by external events | 0 | 1 | 2 | 3 | 4 |
| 306 | I can easily sense when I’m going to be angry | 0 | 1 | 2 | 3 | 4 |
| 307 | I readily tell others my true feelings | 0 | 1 | 2 | 3 | 4 |
| 308 | I find it easy to describe my feelings | 0 | 1 | 2 | 3 | 4 |
| 309 | Even when I’m upset, I’m aware of what’s happening to me | 0 | 1 | 2 | 3 | 4 |
| 310 | I’m able to stand apart from my thoughts and feelings and examine them | 0 | 1 | 2 | 3 | 4 |
|  | B. Self-Management | Never (0) | Rarely (1) | Some times (2) | Often (3) | Always (4) |
| 311 | I accept responsibility for my reactions to them | 0 | 1 | 2 | 3 | 4 |
| 312 | I find it easy to make goals and stick with | 0 | 1 | 2 | 3 | 4 |
| 313 | I am an emotionally balanced person | 0 | 1 | 2 | 3 | 4 |
| 314 | I am a very patient person | 0 | 1 | 2 | 3 | 4 |
| 315 | I can accept critical comments from others without becoming angry | 0 | 1 | 2 | 3 | 4 |
| 316 | I maintain my composure, even during stressful times | 0 | 1 | 2 | 3 | 4 |
| 317 | If an issue does not affect me directly, I don’t let it bother me | 0 | 1 | 2 | 3 | 4 |
| 318 | I can restrain myself when I feel anger towards someone | 0 | 1 | 2 | 3 | 4 |
| 319 | I control urges to overindulge in things that could damage my well-being | 0 | 1 | 2 | 3 | 4 |
| 320 | I direct my energy into creative work or hobbies | 0 | 1 | 2 | 3 | 4 |
|  | C. Social Awareness | Never (0) | Rarely (1) | Some times (2) | Often (3) | Always (4) |
| 321 | I consider the impact of my decisions on other people | 0 | 1 | 2 | 3 | 4 |
| 322 | I can easily tell if the people around me are becoming annoyed | 0 | 1 | 2 | 3 | 4 |
| 323 | I sense it when a person’s mood changes | 0 | 1 | 2 | 3 | 4 |
| 324 | I am able to be supportive when giving bad news to others | 0 | 1 | 2 | 3 | 4 |
| 325 | I am generally able to understand the way other people feel | 0 | 1 | 2 | 3 | 4 |
| 326 | My friends can tell me intimate things about themselves | 0 | 1 | 2 | 3 | 4 |
| 327 | It genuinely bothers me to see other people suffer | 0 | 1 | 2 | 3 | 4 |
| 328 | I usually know when to speak and when to be silent | 0 | 1 | 2 | 3 | 4 |
| 329 | I care what happens to other people | 0 | 1 | 2 | 3 | 4 |
| 330 | I understand when people’s plans change | 0 | 1 | 2 | 3 | 4 |
|  | D. Relationship Management | Never (0) | Rarely (1) | Some times (2) | Often (3) | Always (4) |
| 331 | I am able to show affection | 0 | 1 | 2 | 3 | 4 |
| 332 | I am able to manage relationships well | 0 | 1 | 2 | 3 | 4 |
| 333 | I find it easy to share my deep feelings with others | 0 | 1 | 2 | 3 | 4 |
| 334 | I am good at motivating others | 0 | 1 | 2 | 3 | 4 |
| 335 | I am a fairly cheerful person | 0 | 1 | 2 | 3 | 4 |
| 336 | It is easy for me to make friends | 0 | 1 | 2 | 3 | 4 |
| 337 | People tell me I am sociable and fun | 0 | 1 | 2 | 3 | 4 |
| 338 | I like helping people | 0 | 1 | 2 | 3 | 4 |
| 339 | Others can depend on me | 0 | 1 | 2 | 3 | 4 |
| 340 | I am able to make someone else feel better if they are very upset | 0 | 1 | 2 | 3 | 4 |

Part IV: Leadership effectiveness assessment

| S.No | Items | Scales | | | | |
| --- | --- | --- | --- | --- | --- | --- |
|  |  | Not at all (1) | Once in a while (2) | Some  times (3) | Fairly often (4) | Always (5) |
|  | A. Creating a vision |  |  |  |  |  |
| 401 | I study problems in light of past practices to make sure predictability, strengthen the status quo and minimize risk | 1 | 2 | 3 | 4 | 5 |
| 402 | I feel comfortable in fast-changing environments; being willing to take risks and to consider new and untested approaches | 1 | 2 | 3 | 4 | 5 |
| 403 | I set a clear vision for my sector | 1 | 2 | 3 | 4 | 5 |
| 404 | I communicate the mission and vision of the sector with people around me | 1 | 2 | 3 | 4 | 5 |
| 405 | Taking a long-range, broad approach to problem-solving and decision making through objective analysis, thinking ahead, and planning | 1 | 2 | 3 | 4 | 5 |
|  | B. Developing followership |  |  |  |  |  |
| 406 | I am strongly persuasive and assertive stance to convince my followers | 1 | 2 | 3 | 4 | 5 |
| 407 | I am emotionally expressive and reactive | 1 | 2 | 3 | 4 | 5 |
| 408 | I am ready to develop tomorrow’s leaders | 1 | 2 | 3 | 4 | 5 |
| 409 | I act in an extroverted, friendly, and informal manner; showing a capacity to quickly establish free and easy interpersonal relationships | 1 | 2 | 3 | 4 | 5 |
| 410 | I have ethical values & act consistently | 1 | 2 | 3 | 4 | 5 |
|  | C. Implementing the vision |  |  |  |  |  |
| 411 | I adopt a systematic and organized approach; preferring to work in a precise, methodical manner; developing and utilizing guidelines and procedures | 1 | 2 | 3 | 4 | 5 |
| 412 | I work on a day-to-day basis for achieving the sector vision | 1 | 2 | 3 | 4 | 5 |
| 413 | I emphasize the production of immediate results by focusing on short-range and practical strategies | 1 | 2 | 3 | 4 | 5 |
| 414 | I state clearly what I want and expect from others; clearly, express my thoughts and ideas; maintaining a precise and constant flow of information | 1 | 2 | 3 | 4 | 5 |
| 415 | I capacitate others by giving them important activities and sufficient autonomy to exercise their own judgment. | 1 | 2 | 3 | 4 | 5 |

Part V: Substance use assessments

| S.No | Items |  |  | Remark |
| --- | --- | --- | --- | --- |
| 501 | have you used substances including alcohol currently? | 1. yes  2. No |  | If no, no subsequent questions |
| 502 | Have you drunk alcohol? | 1. Yes  2. No |  | If no, skip to 504 |
| 503 | How often do you drink alcohol? | 1. Every day 2. Twice a week 3. Three times a week 4. Weekly 5. Monthly 6. Others (specify)________ |  |  |
| 504 | Do you chew Khat? |  |  | If no, skip to 506 |
| 505 | How often do you chew Khat? | 1. Every day 2. Twice a week 3. Three times a week 4. Weekly 5. Monthly 6. Others (specify) _______ |  |  |
| 506 | Have you used cigarette smoking? |  |  |  |
| 507 | How often do you smoke cigarettes? | 1. Every day 2. Twice a week 3. Three times a week 4. Weekly 5. Monthly 6. Others (specify) __________ |  |  |

English version semi-structured interview guide

Basic information about the key informant interview (KII) process

Name of the interviewer: _______________

Place/ city: ­­­­­­­­­­­­­­­­­­­­­­­­­­­­­­­­­____________________

Date of interview: _________________

Venue of the interview: _________________

Beginning and ending times of the interview: ___________________

Code number: ___________________

Basic profile of the respondent

Place/city: ___________________

Position: ___________________

Organization: _____________________

Years of experience in that position: _________________

1. How do you describe leadership in this public health institution?
2. How do you describe leadership effectiveness with respect to creating a vision, implementing vision, and developing followership in your public health institution?
3. What do you think the influence of emotional intelligence on creating a vision, implementing vision, and developing followership?
4. How leadership style and managerial level have an influence on creating a vision, implementing vision, and developing followership?
5. Do you think that substance use has an impact on leadership effectiveness? if yes, how?
6. Are socio-demographic characteristics of leaders can influence leadership effectiveness? if yes, how?
7. What are the obstacles in general that hinder leadership effectiveness (creating a vision, implementing vision, and developing followership) in public health institution?
8. What are the facilitators in general that can enhance leadership effectiveness (creating a vision, implementing vision, and developing followership) at public health institution?
